# Immunological Characteristics in Type 2 Diabetes Mellitus Among COVID-19 Patients

**DOI:** 10.3389/fendo.2021.596518

**Published:** 2021-03-11

**Authors:** Meifang Han, Ke Ma, Xiaojing Wang, Weiming Yan, Hongwu Wang, Jie You, Qiuxia Wang, Huilong Chen, Wei Guo, Tao Chen, Qin Ning, Xiaoping Luo

**Affiliations:** ^1^ Department and Institute of Infectious Disease, Tongji Hospital of Tongji Medical College, Huazhong University of Science and Technology, Wuhan, China; ^2^ Department of Radiology, Tongji Hospital, Tongji Medical College, Huazhong University of Science and Technology, Wuhan, China; ^3^ Department of Pediatrics, Center for the Diagnosis of Genetic Metabolic Diseases, Tongji Hospital, Tongji Medical College, Huazhong University of Science and Technology, Wuhan, China

**Keywords:** type 2 diabetes mellitus, COVID-19, immune cells, cytokines, Th1/Th2 ratio

## Abstract

**Clinical Trial Registration:**

www.ClinicalTrials.gov, identifier: NCT04365634.

**Context:**

Diabetes mellitus was associated with increased severity and mortality of disease in COVID-19 pneumonia. So far the effect of type 2 diabetes (T2DM) or hyperglycemia on the immune system among COVID-19 disease has remained unclear.

**Objective:**

We aim to explore the clinical and immunological features of type 2 diabetes mellitus (T2DM) among COVID-19 patients.

**Design and Methods:**

In this retrospective study, the clinical and immunological characteristics of 306 hospitalized confirmed COVID-19 patients (including 129 diabetic and 177 non-diabetic patients) were analyzed. The serum concentrations of laboratory parameters including cytokines and numbers of immune cells were measured and compared between diabetic and non-diabetic groups.

**Results:**

Compared with non-diabetic group, diabetic cases more frequently had lymphopenia and hyperglycemia, with higher levels of urea nitrogen, myoglobin, D-dimer and ferritin. Diabetic cases indicated the obviously elevated mortality and the higher levels of cytokines IL‐2R, IL‐6, IL‐8, IL‐10, and TNF‐α, as well as the distinctly reduced Th1/Th2 cytokines ratios compared with non-diabetic cases. The longitudinal assays showed that compared to that at week 1, the levels of IL-6 and IL-8 were significantly elevated at week 2 after admission in non-survivors of diabetic cases, whereas there were greatly reductions from week 1 to week 2 in survivors of diabetic cases. Compared with survival diabetic patients, non-survival diabetic cases displayed distinct higher serum concentrations of IL-2R, IL-6, IL-8, IL-10, TNF‐α, and lower Th1/Th2 cytokines ratios at week 2. Samples from a subset of participants were evaluated by flow cytometry for the immune cells. The counts of peripheral total T lymphocytes, CD4^+^ T cells, CD8^+^ T cells and NK cells were markedly lower in diabetic cases than in non-diabetic cases. The non-survivors showed the markedly declined counts of CD8^+^ T cells and NK cells than survivors.

**Conclusion:**

The elevated cytokines, imbalance of Th1/Th2 cytokines ratios and reduced of peripheral numbers of CD8^+^ T cells and NK cells might contribute to the pathogenic mechanisms of high mortality of COVID-19 patients with T2DM.

## Introduction

In late December 2019, a novel viral pneumonia with an acute severe respiratory tract illness developed in Wuhan, China, and spread rapidly worldwide, becoming a public health emergency of international concern (1–5). A previously unknown coronavirus, officially named the severe acute respiratory syndrome coronavirus 2 (SARS-CoV-2), with the genome sequence closely related but distinct to severe acute respiratory syndrome or Middle East respiratory syndrome (MERS) virus, was first isolated as a pathogen of the Novel Coronavirus Disease 2019 (COVID-19) by the Chinese Center for Disease Control and Prevention (2, 6, 7).

Our previous data reported the clinical and immunologic features of severe COVID-19 pneumonia (8), indicating that SARS-CoV-2 may primarily affect T lymphocytes, particularly CD4^+^ and CD8^+^ T cells, resulting in a decrease in the number and production of IFN-γ by CD4^+^ T cells (8, 9). Other studies also showed that T cell counts were significantly reduced in patients with COVID-19, and the surviving T cells appeared functionally exhausted (10). An elevated level of cytokines, such as interleukin (IL)-1, IL-2, IL-6, IL-8, IL-10, TNF-α, and soluble IL-2R, and a sharp inflammatory storm have been reported in patients with severe COVID-19 by our team and other researchers (11–16).

The incidence of type 2 diabetes mellitus (T2DM) is increasing worldwide, and diabetes is one of the leading causes of morbidity and mortality globally among chronic diseases (17). Among chronic comorbidities of COVID-19, diabetes had the second highest incidence rate (7.4%–19.0%), following hypertension (15%–30%) (18, 19). Patients with diabetes were likely at higher risk for severe COVID-19 and mortality (20–25). The IL-6, ferritin, C-reaction protein, and D-dimer levels were significantly increased in patients with diabetes, suggesting that a marked inflammatory cytokine storm was associated with a more pejorative prognosis compared to patients without diabetes (22). To date, the detailed effect of diabetes or hyperglycaemia on the immune cells and immune system in patients with COVID-19 remains unclear.

Based on the retrospective study on 306 patients of COVID-19 disease (129 with diabetes and 177 without diabetes), we first identified that patients with diabetes showed distinct immune characteristics from patients without diabetes, including markedly elevated cytokine level, reduced T-helper type 1 (Th1)/T-helper type 2 (Th2) cytokine ratios, and decreased number of CD4^+^ T cells, CD8^+^ T cells, and natural killer (NK) cells. What’s more, we found the elevated cytokines and declined immune cells were associated with the mortality of COVID-19 disease with T2DM. These findings may help broaden our understanding of the pathogenesis mechanism and relationship between diabetes and COVID-19.

## Materials and Methods

### Study Design and Participants

This single-centre, retrospective, observational study was conducted in Tongji Hospital (Wuhan, China), which is a designated hospital for patients with severe COVID-19 pneumonia (NTC04365634). All patients were transported from other hospitals. Patients admitted from February 2, 2020, to February 15, 2020, and diagnosed with COVID-19 pneumonia according to the interim guidance of the National Health Commission of the People’s Republic of China were enrolled in the study (26). Laboratory-confirmed cases were identified with positive viral RNA by real-time reverse transcription polymerase chain reaction (RT-PCR) detection by the local health authority or our hospital from a specimen obtained from a throat or nasal swab. In the enrolled patients, clinical outcomes were evaluated on March 14, 2020. The ethics committee of Tongji Hospital approved the study (TJ-C20200101). Written informed consent was waived due to the rapid emergence of this fatal disease.

### Definitions

Diabetes mellitus was diagnosed according to the standards of the American Diabetes Association (27), which were briefly described as fasting plasma glucose (FPG) level ≥ 7.0 mmol/L (fasting is defined as no caloric intake for at least 8 h) or 2-h plasma glucose level ≥ 11.1 mmol/L during oral glucose tolerance test or with classic symptoms of hyperglycaemia or hyperglycaemic crisis and random plasma glucose (RPG) level ≥11.1 mmol/L. The sepsis-related organ failure assessment (SOFA) and CURB-65 scores are defined or determined using the relative criteria (19, 28, 29). The disease severity of COVID-19 was defined according to the Chinese management guideline for COVID-19 (version 6.0) and described in a previous study (26, 30).

### Laboratory Procedures

Clinical laboratory assays were conducted in the Department of Clinical Laboratory of Tongji Hospital with the certificate of laboratory qualification of China for the assay parameters mentioned above in data collection.

### Real-Time RT-PCR Assay for SARS-CoV-2

The methods of laboratory confirmation of SARS-CoV-2 infection have been described (5, 31). Briefly, SARS-CoV-2 RNA was extracted from throat or nasal swab samples from suspected patients. The presence of SARS-CoV-2 was detected by real-time RT-PCR assay at the local centres of the Chinese Center for Disease Control and Prevention and Tongji Hospital. The SARS-CoV-2 nucleic acid detection kit was used according to the manufacturer’s protocol (DAAN Gene Co., Ltd., of Sun Yat-sen University). These diagnostic methods and criteria were based on the recommendations of the National Institute for Viral Disease Control and Prevention of China.

### Measurements of Cytokines

According to hospital’s standard procedures, fresh blood samples were centrifuged for 10 min at 2,000g. Serum was collected and tested within 4–6 h. All procedures were performed under level 3 protection. Cytokines including interleukin-2 receptor (sIL-2R), IL-1 β, IL-6, IL-8, IL-10, and TNF-α were assessed in serum samples drawn shortly at each time points by chemiluminescence immunoassay (CLIA) performed on a fully automated analyzer (Immulite 1000, DiaSorin Liaison, Italy or Cobas e602, Roche Diagnostics, Germany) for all patients according to the manufacturer’s instructions. IL-2R kit (#LKIP1), IL-1 β kit (#LKL11), IL-8 kit (#LK8P1), IL-10 kit (#LKXP1), and TNF-α kit (#LKNF1) were purchased from DiaSorin (Vercelli, Italy). IL-6 kit (#05109442 190) was purchased from Roche Diagnostics, Germany.

### Number of Peripheral Blood Immunological Cells by Flow Cytometry

Samples from a subset of participants were evaluated by flow cytometry for the immune cells. Fresh peripheral blood samples were obtained from nine patients with and 11 patients without diabetes among the patients with COVID-19 after their agreements, and the proportions and numbers of NK, CD4^+^ T, CD8^+^ T, and B cells and expression of cell surface markers were studied in these patients in a short time. Flow cytometry antibodies against human surface and intracellular molecules were commercially available. The following antibodies were used: BD Multitest 6-Color TBNK reagent (#644611) and BD Trucount Tubes (#340334). It contains FITC-labeled CD3, clone SK7; PE-labeled CD16, clone B73.1, and CD56, clone NCAM16.2; PerCP-Cy5.5-labeled CD45, clone 2D1; PE-Cy7-labeled CD4, clone SK3; APC-labeled CD19, clone SJ25C1; and APC-Cy7-labeled CD8, clone SK1. The BD Trucount™ Absolute Counting Tubes, which contain a known number of fluorescent beads, were used for quantifying leucocyte populations. After another two washes with PBS, cells were resuspended in 500 μl of PBS. Among all collected events, single events were gated between FSC-A and FSC-H. Cell debris was excluded and intact cells were then gated from single events based on FSC-A and SSC. Each cell population was then detected based on the antibody staining. All reagents were purchased from Becton, Dickinson and Company (BD). All samples were detected using the BD FACSCanto II flow cytometry system and analysed using the BD FACSDiva software.

### Chest Computed Tomography (CT) and Evaluation

All chest CT data were acquired using one of the following two commercial multidetector CT scanners: GE Medical Systems/LightSpeed 16 (GE Healthcare, USA) and Siemens/SOMATOM Definition AS (Siemens Healthineers, Germany). Two senior radiologists independently reviewed chest CT images with PACS (Tianjian Health, China). Chest CT images were independently evaluated by two radiologists, and any disagreement in the classification variables was resolved through consultation (32). The distribution of lung abnormalities was mainly subpleural (mainly involving the outer third of the lung), random (subpleural or middle region is not preferred), or diffuse (continuous involvement, not involving the lung segment).

### Statistical Analysis

Continuous variables were presented as median and interquartile range (IQR) and compared using the Mann-Whitney U test. Categorical variables were expressed as numbers (%) and compared using χ^2^ test. Fisher’s exact test was used in the analysis of contingency tables when the sample sizes were small. The Kaplan-Meier method was used for the time-to-event plot in the survival analysis. Comparisons between groups in the survival analysis were conducted using the Cox proportion hazards model. A P-value <0.05 (two-tailed) was considered statistically significant. The data were analysed using SPSS statistical software version 19.0.

## Results

### Demographic and Clinical Characteristics

From February 2, 2020, to February 15, 2020, 306 patients with COVID-19 pneumonia confirmed by positive nucleotide tests of SARS-CoV-2 were enrolled in this study. According to the T2DM criteria, 129 of 306 (42.2%) patients were diagnosed with T2DM and 177 (57.8%) had no T2DM based on FPG or RPG levels (27). The demographic and clinical characteristics on admission are presented in **Table 1**. SOFA and CURB-65 scores, a clinical predictive score for severity of pneumonia, were significantly higher in patients with diabetes compared with those in patients without diabetes. According to the criteria of disease severity in the Chinese management guideline for COVID-19, the proportion of critical illness cases in the diabetic group was higher than that of the non-diabetic group (10.9% vs. 4.5%, p<0.0001), which, together with the SOFA and CURB-65 scores, strongly demonstrated that patients with T2DM tended to have higher disease severity than patients without diabetes.

**Table 1:** Comparing demographics, clinical scores, laboratory findings, and CT image features on admission between T2DM and non-T2DM cases among COVID-19 patients.

	Total	T2DM	Non-T2DM	p value
	n = 306	n = 129	n = 177	
**Demographics and clinical scores**				
Age, years	60.0(49.0–70.0)	65.0(57.0–73.0)	55.0(43.0–67.0)	<0.0001
Gender, Male	174(56.9%)	81(62.8%)	93(52.5%)	0.08
SOFA score	2.0(0.0–3.0)	2.0(1.0–4.0)	1.0(0.0–2.0)	<0.0001
CURB-65 score	1.0(0.0–1.0)	1.0(0.0–2.0)	0.0(0.0–1.0)	<0.0001
Partial arterial oxygen pressure(mmHg)	63.9(44.7–89.8)	58.6(40.8–74.5)	74.5(48.2–117.5)	0.016
Disease severity status*	.	.	.	<0.0001
Moderate	160(52.3%)	51(39.5%)	109(61.6%)	.
Severe	124(40.5%)	64(49.6%)	60(33.9%)	.
Critical	22(7.2%)	14(10.9%)	8(4.5%)	.
**Laboratory findings**				
Fasting plasma glucose(mmol/L)	6.3(5.4–8.7)	9.4(7.7–13.6)	5.6(5.1–6.2)	<0.0001
<7.0	174/274(63.5%)	14/110(12.7%)	160/164(97.6%)	<0.0001
7.0–11.1	60/274(21.9)	56/110(50.9%)	4/164(2.4%)	<0.0001
≥11.1	40/274(14.6%)	40/110(36.4%)	0/164(0.0%)	<0.0001
Random plasma glucose(mmol/L)	6.7(5.7–8.9)	8.9(7.2–14.1)	6.0(5.4–6.8)	<0.0001
<7.0	158(53.9%)	27(20.9%)	138(78.0%)	<0.0001
7.0–11.1	94(30.7%)	57(44.2%)	37(20.9%)	<0.0001
≥11.1	47(15.4%)	45(34.9%)	2(1.1%)	<0.0001
White blood cell count(10^9^/L)	5.6(4.4–8.0)	6.6(4.7–10.1)	5.1(4.3–7.2)	<0.0001
>10 (ULN)	50(16.3%)	33(25.6%)	17(9.6%)	<0.0001
Lymphocyte count(10^9^/L)	0.8(0.6–1.2)	0.7(0.5–0.9)	0.9(0.7–1.3)	<0.0001
<1.1 (LLN)	213(69.6%)	106(82.2%)	107(60.5%)	<0.0001
ALB(g/L)	33.4(30.4–36.6)	32.4(29.6–35.8)	34.2(31.4–37.4)	0.002
≤30 (LLN)	67/303(22.1%)	38/128(29.7%)	29/175(16.6%)	0.007
LDH(U/L)	324.0(246.0–451.0)	361.0(266.3–490.8)	306.0(237.0–414.0)	0.001
>225 (ULN)	249/303(82.2%)	107/128(83.6%)	142/175(81.1%)	0.58
Total cholesterol (mmol/L)	3.5(3.0–4.0)	3.4(3.0–4.1)	3.5(3.0–4.0)	0.67
>5.18 (ULN)	7/303(2.3%)	5/128(3.9%)	2/175(1.1%)	0.11
Triglyceride (mmol/L)	1.3(1.0–1.8)	1.3(1.0–1.9)	1.3(1.0–1.8)	0.55
>1.7 (ULN)	40/139(28.8%)	21/68(30.9%)	19/71(26.8%)	0.59
urea nitrogen (mmol/L)	4.7(3.5–7.0)	5.6(3.9–9.0)	4.3(3.1–5.3)	<0.0001
>8 (ULN)	56/303(18.5%)	39/128(30.5%)	17/175(9.7%)	<0.0001
D-dimer (μg/ml)	1.0(0.5–2.6)	1.6(0.7–7.0)	0.7(0.4–1.6)	<0.0001
≥0.5 (ULN)	215/287(74.9%)	105/124(84.7%)	110/163(67.5%)	0.001
High-sensitivity cardiac troponin I (pg/ml)	7.3(2.8–20.7)	15.6(5.2–41.5)	3.9(2.2–13.3)	<0.0001
>28 (ULN)	45/218(20.6%)	32/99(32.3%)	13/119(10.9%)	<0.0001
Myoglobin (ng/ml)	93.1(34.4–183.1)	128.0(59.5–244.7)	32.0(22.7–104.0)	0.003
>106 (ULN)	21/45(46.7%)	17/29(58.6%)	4/16(25.0%)	0.03
PCT(ng/ml)	0.1(0.04–0.2)	0.1(0.1–0.3)	0.1(0.03–0.2)	<0.0001
≥0.05 (ULN)	186/289(64.4%)	94/120(78.3%)	92/169(54.4%)	<0.0001
CRP (mg/L)	53.0(20.5–101.4)	79.6(35.3–137.5)	44.6(18.2–84.9)	0.002
≥1	301/302(99.7%)	86/86(100.0%)	215/216(99.5%)	1.000
Serum ferritin (μg/L)	885.0(488.5–1566.2)	1086.5(571.3–1809.6)	652.5(380.2–1347.1)	0.002
>300 (ULN)	137/155(88.4%)	79/82(96.3%)	58/73(79.5%)	0.001
**Cytokines**				
IL-1B (pg/ml)	5.0(5.0–5.0)	5.0(5.0–5.0)	5.0(5.0–5.0)	0.27
≥5 (ULN)	241/242(99.6%)	106/106(100.0%)	135/136(99.3%)	1.00
IL-2R (μ/ml)	740.0(528.3–1063.3)	925.5(567.5–1256.3)	676.0(475.0–911.8)	<0.0001
>710 (ULN) or <223 (LLN)	130/242(53.7%)	67/106(63.2%)	63/136(46.3%)	0.009
IL-6(pg/ml)	16.4(3.9–54.4)	31.5(6.1–79.0)	12.7(2.7–35.5)	0.001
≥7 (ULN)	158/242(65.3%)	78/106(73.6%)	80/136(58.8%)	0.017
IL-8 (pg/ml)	15.1(8.8–24.3)	19.8(10.6–30.4)	12.0(8.2–21.3)	<0.0001
≥62 (ULN)	18/242(7.4%)	13/106(12.3%)	5/136(3.7%)	0.012
IL-10 (pg/ml)	5.3(5.0–10.0)	6.8(5.0–14.8)	5.0(5.0–8.4)	0.001
≥9.1 (ULN)	71/242(29.3%)	42/106(39.6%)	29/136(21.3%)	0.002
TNF-α (pg/ml)	8.4(6.8–10.8)	9.2(7.1–12.5)	8.1(6.2–9.8)	0.001
≥8.1 (ULN)	134/242(55.4%)	66/106(62.3%)	68/136(50.0%)	0.06
**CT image features**				
Distribution of pulmonary lesions	.	.	.	0.15
Peripheral	42/185(22.7%)	11/67(16.4%)	31/118(26.3%)	.
Random	30/185(16.2%)	9/67(13.4%)	21/118(17.8%)	.
Diffuse	113/185(61.1%)	47/67(70.1%)	66/118(55.9%)	.
Bilateral multilobe, n	177,185(95.7%)	66/67(98.5%)	111/118(94.1%)	0.26
Ground-glass opacity (GGO), n	176/185(95.1%)	64/67(95.5%)	112/118(95.1%)	1.00
Crazy-paving pattern, n	106/185(57.3%)	37/67(55.2%)	69/118(58.5%)	0.76
Consolidation, n	152/185(82.2%)	58/67(86.6%)	93/118(78.8%)	0.24

Data are median (IQR), n (%), or n/N (%). p values were calculated by Mann-Whitney U test, χ² test, or Fisher’s exact test, as appropriate. T2DM, type 2 diabetes mellitus; ALB, albumin; TBil, Total bilirubin; DBil, Direct bilirubin; LDH, Lactate dehydrogenase; PCT, Procalcitonin; ULN, Upper limit of normal value; LLN, lower limit of normal value. * Disease severity status was evaluated according to interim guidance of National health commission of China.

### Laboratory Parameters and Clinical Outcomes

There were strong differences in laboratory findings on admission between patients with diabetes and those without diabetes (**Table 1**). The FPG and RPG levels were distinctly higher in the diabetes group than in the non-diabetes group. The peripheral lymphocyte count was significantly lower in patients with diabetes than in those without diabetes (p<0.0001), whereas the white and neutral cell counts were higher in patients with diabetes than in those without diabetes, which might be related to the higher risk of infection in patients with diabetes. Laboratory parameters, including the serum urea nitrogen, high-sensitivity cardiac troponin I, myoglobin, D-dimer, lactate dehydrogenase, procalcitonin, C-reactive protein, and ferritin levels, were markedly higher in patients with diabetes than in those without diabetes. However, there was no significant difference in the total cholesterol and triglyceride levels between the diabetes and non-diabetes groups. With regard to the lung CT features, the diabetic group showed no distinct difference from the non-diabetic group.

**Figure 1:**
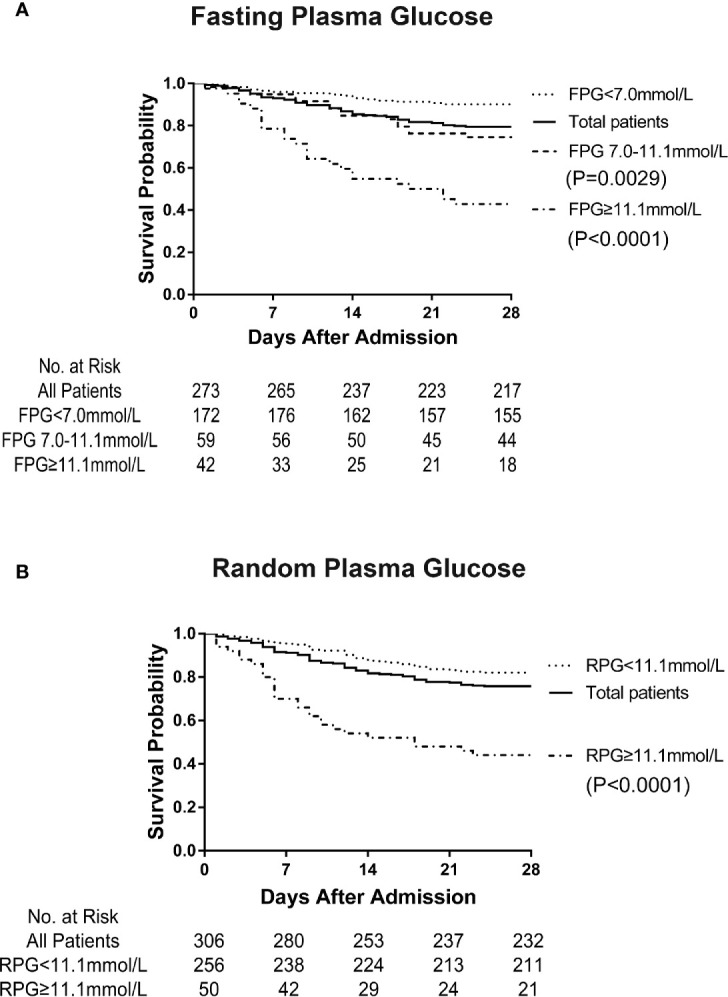
Survival curve of patients with COVID-19 affected by fasting plasma glucose (FPG) and random plasma glucose (RPG) levels on admission. It shows the greatly decreased survival in patients with FPG level ≥ 7.0 mmol/L on admission compared with that in patients with FPG level < 7.0 mmol/L (p < 0.0001) **(A)**. It indicates the obviously decreased survival in patients with RPG level ≥11.1 mmol/L on admission compared with that in patients with RPG level <11.1 mmol/L (p < 0.0001) **(B)**.

Our data showed that the mortality on the 28th day after admission in the hospital was much higher in patients with diabetes than in those without diabetes (42.6% vs. 10.7%, p<0.0001). As shown in **Figure 1**, the Kaplan-Meier survival curves were affected by different FPG and RPG levels (**Figures 1A, B**). Patients with FPG level ≥ 11.1 mmol/L (p<0.0001) and FPG level between 7 and 11.1 mmol/L (p=0.0029) showed a greatly decreased survival compared to patients with FPG level < 7.0 mmol/L (separately) (**Figure 1A**). Patients with RPG level ≥ 11.1 mmol/L showed much decreased survival curves than patients with RPG level < 11.1 mmol/L (p<0.0001) (**Figure 1B**). With the increased FPG or RPG levels, the rates of death of patients with COVID-19 were raised accordingly, thus suggesting that hyperglycaemia might be an important risk factor for the mortality of patients with COVID-19.

### Markedly Elevated Cytokine Levels and Decreased Th1/Th2 Cytokine Ratios in Patients With Diabetes

**Figure 2:**
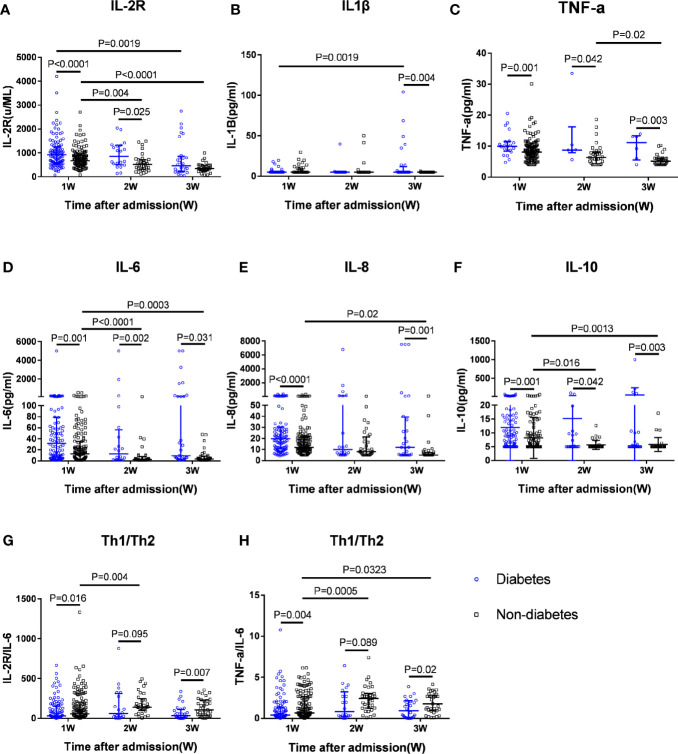
Dynamic serum cytokine levels and Th1/Th2 ratios in diabetic patients and non-diabetic patients. Dynamic changes in Th1 cytokines including IL-2R **(A)**, IL-1β **(B)**, and TNF-α **(C)**; Th2 cytokines, including IL-6 **(D)**, IL-8 **(E)**, and IL-10 **(F)**, and ratios of IL-2R/IL-6 **(G)** and TNF-α/IL-6 **(H)** representing the Th1/Th2 ratios from week 1 to week 3 after admission in patients with T2DM and patients without T2DM with COVID-19 infection.

Regarding the Th1 cytokines, patients with diabetes indicated markedly higher IL-2R levels in week 1 and 2 after admission, IL-1β in week 3, and TNF-α in week 1–3 compared to patients without diabetes (**Figure 2**). As for the Th2 cytokines, the IL-6 and IL-10 levels in patients with diabetes in week 1–3, and IL-8 in week 1–3 were significantly higher than those in patients without diabetes. IL-6 and IL-10 levels in the patients without diabetes were observed to be reduced successively from week 1 to 3 and greatly decreased at week 2 and 3 compared to that in week 1, whereas they were not reduced in patients with diabetes from week 1 to week 3. These results indicated that patients with diabetes had significantly elevated inflammatory cytokine Th1 and Th2 levels, which is known as a cytokine storm.

Furthermore, we used the IL-2R/IL-6 and TNF-α/IL-6 ratios on behalf of the Th1/Th2 ratios to determine which type of T-helper cells was dominant in the progression of COVID-19. The Th1/Th2 ratios in patients with diabetes were much lower than in patients without diabetes through weeks 1–3 after admission, suggesting that Th2 cells were over-activated and the imbalance of Th1/Th2 cytokines in patients with diabetes was much greater than in patients without diabetes. Moreover, in patients without diabetes, the IL-2R/IL-6 and TNF-α/IL-6 ratios increased greatly in week 2 compared to that in week 1 (P=0.004 and P=0.0005, respectively), indicating that the imbalance of Th1/Th2 ratios recovered to some degree in week 2 in patients without diabetes, while there was no difference in patients with diabetes between week 1 and week 2. Thus, patients with diabetes showed longer imbalance of Th1/Th2 than in patients without diabetes.

### Greatly Increased Levels of Cytokines in Non-Survival Diabetic COVID-19 Cases

**Figure 3:**
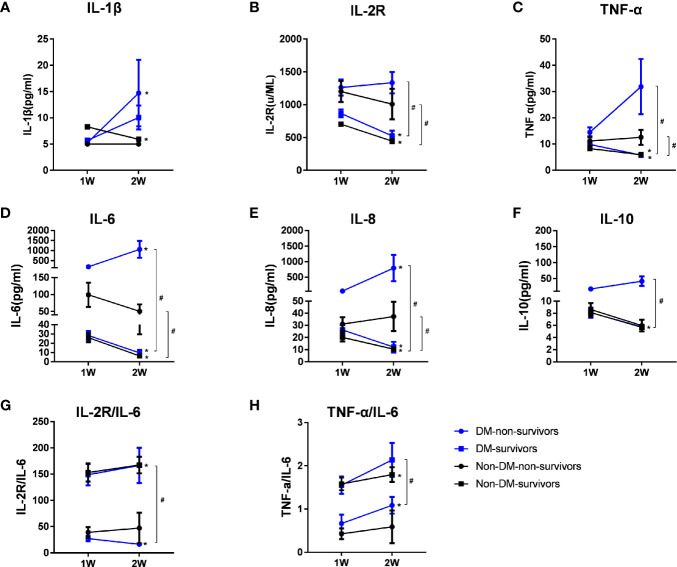
Dynamic changes of serum cytokine levels and Th1/Th2 ratios in groups of diabetic non-survivors, diabetic survivors, non-diabetic non-survivors and non-diabetic survivors from week 1 to week 2. Dynamic changes in Th1 cytokines including IL-1β **(A)**, IL-2R **(B)**, and TNF-α **(C)**; Th2 cytokines, including IL-6 **(D)**, IL-8 **(E)**, and IL-10 **(F)**, and ratios of IL-2R/IL-6 **(G)** and TNF-α/IL-6 **(H)** representing the Th1/Th2 ratios from week 1 to week 2 after admission in survivors and non-survivors with COVID-19 infection. Data shown are median (IQR). *represents P < 0.05, week 2 vs. week 1. # represents P < 0.05, non-survivors vs. survivors at week 2 in diabetic or non-diabetic patients. DM, diabetes mellitus.

To assess the association between the mortality of COVID-19 and the levels of cytokines, we analyzed the longitudinal expression profiles of cytokines from week 1 to week 2 after admission in survivors and non-survivors (**Figure 3**). Compared to that at week 1, the levels of IL-6 and IL-8 were significantly elevated at week 2 in non-survivors of diabetic cases, whereas there were greatly reductions in survivors of diabetes at week 2 than at week 1 (P<0.05, respectively). The ratios of IL-2R/IL-6 in diabetic non-survivors at week 2 were much declined compared to that at week 1, whereas they were lifted greatly in non-diabetic survivors, or there were no significant changes in the diabetic survivors and non-diabetic non-survivors from week 1 to week 2.

Compared with diabetic survival patients, diabetic non-survival cases showed distinct higher serum concentrations of IL-6, IL-8 and TNF‐α and lower Th1/Th2 cytokines ratios (IL-2R/IL-6 and TNF-α/IL-6) at week 2 (P<0.05, respectively), suggesting that these higher levels of cytokines and much greater imbalance of Th1/Th2 cytokines ratios might be involved in the pathogenic mechanisms of mortality for diabetic COVID-19 patients. The median values of cytokines and P values of comparing the levels of cytokines among different groups including diabetic non-survivors, diabetic survivors, non-diabetic non-survivors and non- diabetic survivors patients were displayed in **Supplementary Table 1**.

### Greatly Decreased Numbers of CD4^+^ T Cells, CD8^+^ T Cells, and NK Cells in Diabetic Cases

**Figure 4:**
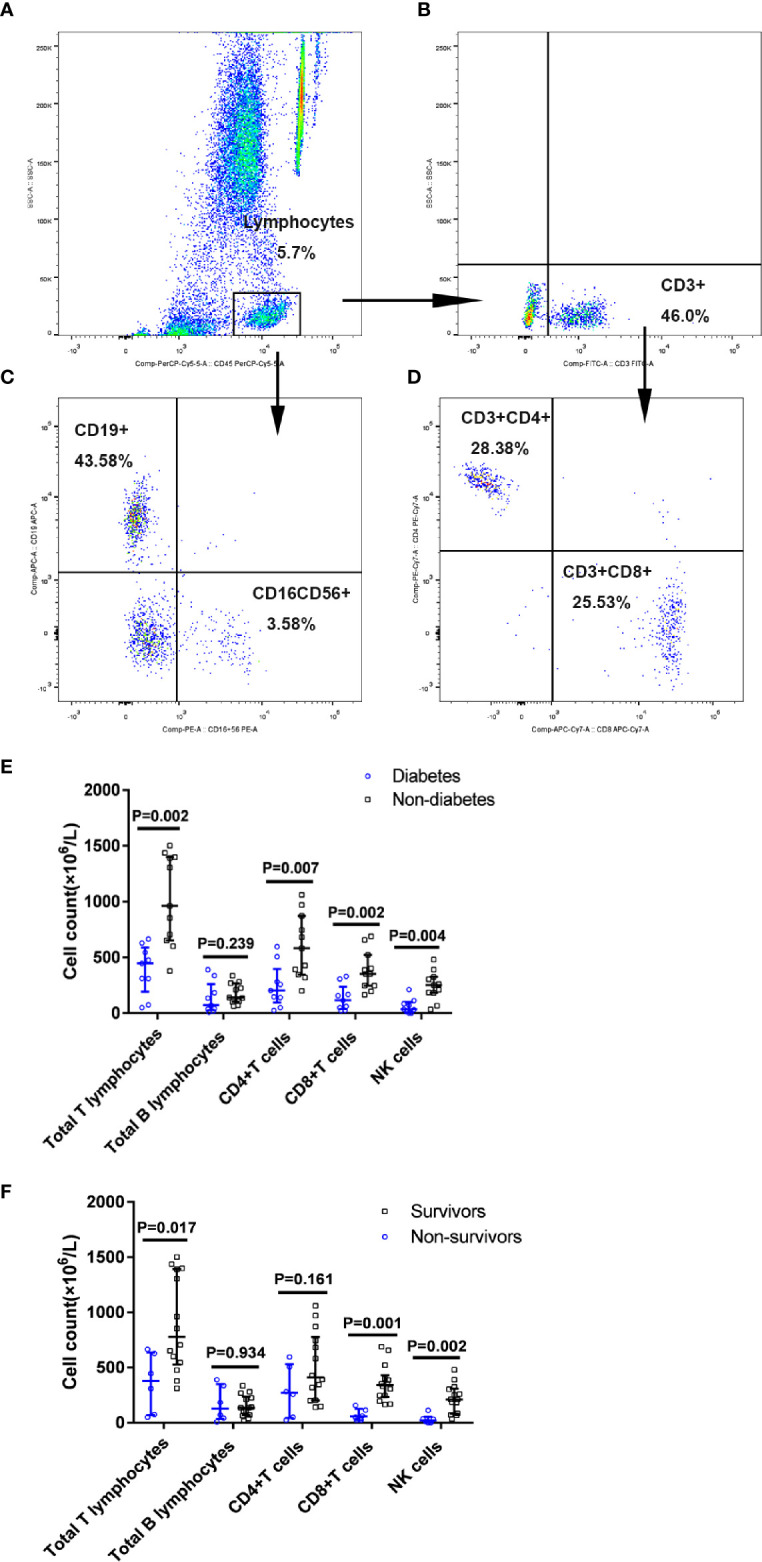
Proportion of immune cells subsets in patients with diabetes with COVID-19. Flow cytometric analysis of B cells, NK cells, CD4+ T cells, and CD8+ T cells from a representative patient **(A–D)**. A series of comparisons of absolute counts of total T and B lymphocytes, CD4^+^ T cells, CD8^+^ T cells, and NK cells between patients with diabetes (n=9) and patients without diabetes (n=11) **(E)**, as well as between survivors (n=14) and non-survivors (n=6) **(F)**. All data represent median (IQR). Differences were tested using nonparametric test.

Then, we examined the proportion and counts of immune cells in the peripheral blood from nine patients with diabetes and 11 patients without diabetes among the patients with COVID-19 (**Table 2** and **Figure 4**). It was found that absolute numbers of total T lymphocytes were more seriously reduced in the patients with diabetes compared to those in patients without diabetes (448.0 vs. 962.0 × 10^6^/L, P=0.002), while the absolute numbers of total B lymphocytes did not differ between the two groups. Furthermore, the numbers of CD4^+^ T cells and CD8^+^ T cells were reduced below the lower limit of normal (LLN) in the vast majority of patients with diabetes with COVID-19, and the medians of the diabetic group were reduced more profoundly than in the non-diabetic group (204.0 vs. 583.0 ×10^6^/L, P=0.007 and 115.0 vs. 352 ×10^6^/L, P=0.002, respectively). As for the NK cell count, it could be known that a lower NK cell count was observed in patients with diabetes compared to that in patients without diabetes (35.0 vs 252.0×10^6/^L, P=0.004) (**Figure 4E**). Moreover, we found a greater reduction in the total absolute numbers of T, B, and NK cells in the diabetic group than in the non-diabetic group (P=0.002). In addition, all diabetic cases showed a significant decrease in total T lymphocyte counts < 955 × 10^6/^L (LLN), CD8^+^ T cell counts < 320 × 10^6^/L (LLN), and NK cell counts < 150 × 10^6^/L (LLN). Of 9 patients with diabetes, 7 (77.8%) showed obvious broad decrease in all lymphocyte subsets, including total B cell count < 90 × 10 ^6^/L, CD4^+^ T cell count < 550 × 10^6/^L, and the abovementioned total T lymphocyte, CD8^+^ T cell, and NK cell counts. Of these nine diabetic patients, 3 (33.3%) eventually died, and an image of the flow cytometry of one dead patient is shown in **Figures 4A–D**. The proportion of NK cells (3.58%) obviously decreased.

**Table 2:** Comparing the counts and frequencies of immune cells on admission between T2DM and non-T2DM cases.

	Total	T2DM	Non-T2DM	p value
	n = 20	n = 9	n = 11	
Total T lymphocytes (%)	70.4(56.6–76.2)	69.0(53.7–75.7)	71.4(60.1–76.9)	0.569
Total T lymphocytes count (10^6^/L)	640.5(396.3–1219.3)	448.0(193.0–587.0)	962.0(652.0–1,399.0)	0.002
Decreased, <955, n/N (%)	14(70.0%)	9(100.0%)	5(45.5%)	0.014
<400, n/N (%)	5(25.0%)	4(44.4%)	1(9.1%)	0.127
Total B lymphocytes (%)	17.2(8.9–19.7)	19.9(13.4–36.4)	13.3(7.9–17.5)	0.025
increased, n/N (%)	8(40.0%)	6(66.7%)	2(18.2%)	0.065
Total B lymphocytes count (10*6/L)	135.5(65.5–258.3)	72.0(27.0–261.0)	141.0(103.0–269.0)	0.239
decreased, n/N (%)	7(35.0%)	5(55.6%)	2(18.2%)	0.160
CD4+T cells (%)	39.0(27.7–50.3)	29.5(25.7–58.3)	39.5(28.6–48.9)	0.970
CD4+T cells count (10^6^/L)	368.5(201.0–661.8)	204.0(95.0–395.5)	583.0(345.0–872.0)	0.007
decreased, n/N (%)	13(65.0%)	8(88.9%)	5(45.5%)	0.070
CD8+T cells (%)	22.2(17.3–36.5)	23.6(13.2–36.4)	21.7(20.1–37.8)	0.569
CD8+T cells count (10^6^/L)	248.0(125.8–379.0)	115.0(38.0–238.0)	352.0(248.0–522.0)	0.002
decreased, n/N (%)	12(60.0%)	8(88.9%)	4(36.4%)	0.028
NK cells (%)	11.4(4.6–23.67)	10.3(3.5–12.3)	14.4(10.2–28.9)	0.074
NK cells count (10^6^/L)	148.0(34.3–258.8)	35.0(7.5–99.5)	252.0(182.0–326.0)	0.004
Decreased, <150, n/N (%)	10(50.0%)	8(88.9%)	2(18.2%)	0.005
<77, n/N (%)	8(40.0%)	6(66.7%)	2(18.2%)	0.065
T+B+NK(%)	99.3(98.9–99.5)	99.3(98.9–99.4)	99.4(98.7–99.6)	0.761
T+B+NK(#)	984.5(695.3–1,693.5)	685.0(241.0–899.0)	1584.0(1016.0–1795.0)	0.002
Th/Ts	1.6(0.9–2.4)	1.8(0.7–4.8)	1.4(1.0–2.4)	0.970

Among these 20 COVID-19 patients, there were six non-survivors including three diabetic and three non-diabetic patients and 14 survivors including six diabetic and eight non-diabetic patients. When compared with the survivors, non-survivors displayed the greater reductions in the absolute numbers of total T lymphocytes, CD8^+^ T cells and NK cells (P<0.05) (**Figure 4F**). The proportion and counts of immune cells in the peripheral blood from six non-survivors and 11 survivors with COVID-19 were showed in **Supplementary Table 2**.

## Discussion

Patients with COVID-19 with T2DM are likely to develop a severe form of the disease (22). In this retrospective cohort with 306 severe patients with SARS-CoV-2 infection, including 129 patients with diabetes and 177 patients without diabetes, we found that the patients with diabetes had much higher mortality rates (42.6%) than patients without diabetes (10.7%) and first proved that patients with FPG level > 7.0 mmol/L or RPG level > 11.1 mmol/L on admission have a significantly decreased chance of survival, which profoundly indicates that diabetes or hyperglycaemia might be a potential risk factor of fatality in COVID-19. To further investigate the reason for this high mortality, we reviewed the immune status of patients and found that T2DM showed markedly reduced numbers of immune cells, such as CD4^+^, CD8^+^ T, NK cells, and obvious imbalance of Th1/Th2 cytokine signalling over-activated Th2 cell function, thus aggravating the severity of COVID-19.

This study reports that patients with T2DM have higher SOFA and CURB-65 scores than patients without diabetes, suggesting that higher rates of multiple organ failure and fatal pneumonia lead to higher mortality in patients with T2DM. Diabetes, with its high morbidity and mortality, has grown to a global health problem in recent decades, owing to increasing risks of infection, cardiovascular disease, and other diseases (17). Recently, a retrospective, multicentered study of COVID-19 found that patients with T2DM required more medical interventions and had a significantly higher mortality rate (7.8% vs. 2.7%) (33). The relationship between diabetes and infection has long been recognised (34). Infections, particularly influenza and pneumonia, are often common and more serious in elderly patients with T2DM (35). T2DM has been recognised as a risk factor for disease progression and mortality in SARS-CoV, MERS-CoV, and novel SARS-CoV-2 infections (22, 36–41).

Patients with T2DM with higher plasma glucose level (FPG level > 7 mmol/L or RPG level > 11.1 mmol/L) in our study were demonstrated to have a greatly decreased survival compared to patients with FPG level < 7.0 mmol/L or RPG level < 11.1 mmol/L, separately. Similar to this conclusion, a recent study indicated that a well-controlled blood glucose level (3.9 to 10.0 mmol/L) was associated with markedly lower mortality rate compared to poorly controlled blood glucose level (> 10.0 mmol/L) during hospitalisation. A meta-analysis showed that diabetes was associated with poor outcomes, including mortality, severity status, acute respiratory distress syndrome, need for intensive care, and disease progression (42). Recently, a large national investigation in England show that type 1 and type 2 diabetes were both independently associated with a significant increased odds of in-hospital death with COVID-19, which supported our conclusions furtherly (43) and BMI was identified to be independently associated with the severity of COVID-19 in French CORONADO study (44).

So far, there has been scarce data regarding the relationship between glucose metabolism and immune response in patients with COVID-19. In this study, we first presented markedly elevated Th1 cytokine IL-2R and TNF-α levels and increased levels of Th2 cytokines, including IL-6, IL-8, and IL-10, in patients with diabetes compared with patients without diabetes among patients with COVID-19 pneumonia. Our results provide novel evidence that the imbalance of Th1/Th2 cytokines, a significant decrease in the Th1/Th2 cytokine ratio in patients with diabetes, suggested the over-activation of Th2 cells, which may account for the disturbance of the immune system, causing poor prognosis. We furtherly found that the lifted expression levels of cytokines were associated with the mortality of COVID-19 patients with T2DM. Non-survivors of diabetic cases showed the significant increases of IL-6 and IL-8 at week 2 compared to that at week 1, whereas there were greatly reductions in survivors of diabetes cases. The ratios of IL-2R/IL-6 in diabetic non-survivors at week 2 were much declined compared to that at week 1, whereas they were raised greatly in non-diabetic survivors, and there were no significant changes in the survivors with or without T2DM from week 1 to week 2. The data indicated that elevated cytokines and greater imbalance of Th1/Th2 ratios might be involved the immune pathogenic mechanisms of COVID-19 patients with T2DM and lead to higher mortality compared with non-diabetic patients.

To the best of our knowledge, Th2 cells typically produce IL-4, IL-6, Il-8, IL-10, and IL-13, whereas cytokines, such as IL-1β, IL-2R, and TNF-α, belong to the Th1 cell response. As two extremes on a scale, Th1 and Th2 responses play different roles and may contribute to immunopathology. Distinct from Th1 cell pro-inflammatory function and antiviral response by stimulating macrophages and cell-mediated immunity, Th2 cells tend to oppose the inflammatory reaction and promote antibody response and inhibit Th1 cell-induced antiviral function (45). Under normal conditions, IFN-γ can induce the differentiation of Th0 cells into Th1 cells, whereas, during SARS-CoV-2 infection, the lower level of IFN-γ production would reduce the production of Th1, resulting in further weakening of the antiviral immune response of CD4^+^ T cells. In addition, T2DM is a chronic inflammatory condition characterised by multiple metabolic and vascular abnormalities that can affect the immune response to pathogens (46). Both cytokine disturbances and T-cell exhaustion in patients with diabetes indicated poor clinical outcomes (10, 12, 16, 47–49). Hyperglycaemia and insulin resistance promote increased synthesis of glycosylation end products and pro-inflammatory cytokines, oxidative stress, and adhesion molecules, which may be the underlying mechanism that leads to a higher propensity to infections, with worse outcomes in patients with diabetes (50). Targeting the overexpression of IL-6 effects with a monoclonal antibody against IL-6 receptor or using Janus Kinase inhibitors may be particularly helpful for treatment of COVID-19 pneumonia in diabetes in the future (51). A previous study found Th1 cytokines including IFN-γ, TNF-α, and CRP but not Th2 cytokines appeared significantly higher in the T2DM group than in the non-T2DM group. Unlike the above study, we found both Th1 and Th2 cytokines were greatly elevated in T2DM compared with non-T2DM when infected with SARS-CoV-2 (52).

Our study reported that patients with diabetes present a greatly reduced number of lymphocytes, especially the decreased counts of peripheral CD4^+^ T cells, CD8^+^ T cells, and NK cells compared to patients without diabetes in SARS-CoV-2 infections. Guo et al.’s study on viral pneumonia, such as influenza A, adenovirus, bocavirus, human rhinovirus, and coronavirus, but not SARS-CoV-2, revealed a higher mortality rate in patients with lower absolute counts of CD8^+^ T and CD4^+^ T cells (53). As for COVID-19 pneumonia, T cell counts are reduced significantly in patients with COVID-19, and the surviving T cells appear functional exhausted (54, 55).

SARS-CoV infection can significantly reduce peripheral CD4^+^ and CD8^+^ T lymphocyte subsets, which is related to the onset of the disease (56). Similarly, MERS-CoV could effectively induce apoptosis of T cells in the peripheral blood and human lymphoid organs, involving activation pathways of extrinsic and intrinsic apoptosis (57). Presumably, infection of epithelial cells in the airways and subsequent replication of the virus in these tissues might cause high levels of virus-linked apoptosis or pyroptosis, triggering inflammatory responses marked by the activation of pro-inflammatory cytokines or chemokines. Based on recent clinical data obtained from COVID-19 patients, cytokine storm, pulmonary, and endothelial dysfunction, and hypercoagulation condition may contributes to pathogenic mechanisms of COVID-19 patients with T2DM (58). Hyperglycaemia can damage the hypothalamic–pituitary–adrenal axis, resulting in high cortisol secretion. The over-secreted cortisol might not only elevate the serum glucose level but also suppress the immune system and immune cells, leading to a vicious circle (59).

Our study provides distinct evidence that T2DM or hyperglycaemia patients showed an obvious decrease in immune cells and imbalance of TH1/Th2 cytokines, which were associated with the high mortality of COVID-19 patients with T2DM.

## Conclusions

In COVID-19, T2DM or hyperglycaemia affected the numbers of immune cells, including CD4^+^, CD8^+^ T cells, and NK cells, and reduced Th1/Th2 cytokine ratios, which might aggravate the severity of COVID-19. This study may shed light on the complex immunological mechanisms and relationship between T2DM and COVID-19.

## Data Availability Statement

The original contributions presented in the study are included in the article/**Supplementary Material**. Further inquiries can be directed to the corresponding authors.

## Ethics Statement

The studies involving human participants were reviewed and approved by the Ethical Committee of Tongji Hospital, Tongji Medical College, and Huazhong University of Science and Technology. Written informed consent for participation was not required for this study in accordance with the national legislation and the institutional requirements.

## Author Contributions 

All authors have been fully involved in the preparation of the manuscript at all stages and approved it for publication. All authors contributed to the article and approved the submitted version.

## Funding

This work is funded by grants from Tongji Hospital for Pilot Scheme Project, and partly supported by the

Chinese National Thirteenth Five Years Project in Science and Technology (2017ZX10202201).

## Conflict of Interest

The authors declare that the research was conducted in the absence of any commercial or financial relationships that could be construed as a potential conflict of interest.

## Abbreviations

PHEIC, Public health emergency of international concern; SARS-CoV-2, Severe acute respiratory syndrome coronavirus 2; COVID-19, Coronavirus Disease 2019; T2DM, Type 2 diabetes mellitus; SOFA, Sepsis-related organ failure assessment; GGO, Ground-glass opacity; Th1, T-helper type 1; Th2, T-helper type 2; RPG, Random plasma glucose; FPG, Fasting plasma glucose; ULN, Upper limit of normal value; LLN, Lower limit of normal value; IQR, Interquartile range.
