# Supplementary material for: Immunological Characteristics in Type 2 Diabetes Mellitus Among COVID-19 Patients
Source: Front Endocrinol (Lausanne). 2021 Mar 11;12:596518. doi: 10.3389/fendo.2021.596518 (PMC7992040; doi:10.3389/fendo.2021.596518)
Supplement: Supplementary file 1 [file Table_1.docx]

**Supplementary Table 1. Median values of serum cytokines and Th1/Th2 ratios in groups of diabetic non-survivors, diabetic survivors, non-diabetic non-survivors and non-diabetic survivors at week 1 and week 2, as well as the P values comparing the levels of cytokines in the different groups.**

|  | Week 1 | | | | Week 2 | | | | | P | | | | | | | |
| --- | --- | --- | --- | --- | --- | --- | --- | --- | --- | --- | --- | --- | --- | --- | --- | --- | --- |
|  | 1 | 2 | 3 | 4 | | 5 | 6 | 7 | 8 | 1 vs 5 | 2 vs 6 | 3 vs 7 | 4 vs 8 | 5 vs 6 | 7 vs 8 | 5 vs 7 | 6 vs 8 |
| IL-1β(pg/ml) | 5 | 5 | 5 | 8.05 | | 5 | 5 | 5 | 5.7 | 0.0104 | 0.195 | >0.9999 | <0.0001 | 0.406 | 0.5 | 0.5166 | 0.9447 |
| IL-2R(u/ML) | 1136 | 729 | 1062 | 661 | | 1257 | 410 | 1035 | 394 | 0.472 | <0.0001 | 0.721 | <0.0001 | <0.0001 | 0.0002 | 0.4845 | 0.8766 |
| TNF-α(pg/ml) | 10.1 | 8.9 | 8.3 | 8.05 | | 13.15 | 5 | 12.1 | 5.7 | 0.0802 | <0.0001 | 0.431 | <0.0001 | <0.0001 | <0.0001 | 0.5288 | 0.8885 |
| IL-6(pg/ml) | 59.76 | 9.3 | 47.3 | 11.4 | | 157.4 | 3.93 | 40.85 | 2.485 | 0.0243 | 0.003 | 0.878 | <0.0001 | <0.0001 | <0.0001 | 0.0804 | 0.2424 |
| IL-8(pg/ml) | 23.55 | 17 | 25.9 | 11.7 | | 94 | 6.15 | 41.5 | 5.9 | 0.0009 | <0.0001 | 0.659 | <0.0001 | <0.0001 | <0.0001 | 0.1995 | 0.8271 |
| IL-10(pg/ml) | 13.65 | 5 | 8.1 | 5 | | 9.85 | 5 | 5 | 5 | 0.9416 | 0.055 | 0.178 | 0.0007 | 0.0001 | 0.797 | 0.0607 | 0.2515 |
| IL-2R/IL-6 | 19.1 | 96 | 35 | 77.6 | | 7.149 | 111 | 25.58 | 142.7 | 0.0376 | 0.582 | 0.878 | 0.02 | <0.0001 | 0.878 | 0.2269 | 0.3908 |
| TNF-α/IL-6 | 0.22 | 1 | 0.27 | 0.78 | | 0.84 | 1.31 | 0.291 | 1.242 | 0.0022 | 0.079 | 0.645 | 0.007 | 0.015 | 0.081 | 0.3414 | 0.952 |

Note:

Group 1 = DM-non-survivors at week 1 (n=44)

Group 2 = DM-survivors at week 1 (n=62)

Group 3 = Non-DM-non-survivors at week 1 (n=14)

Group 4 = Non-DM-survivors at week 1(n=122)

Group 5 = DM-non-survivors at week 2 (n=18)

Group 6 = DM-survivors at week 2 (n=30)

Group 7 = Non-DM-non-survivors at week 2 (n=4)

Group 8 = Non-DM-survivors at week 2 (n=62)

DM, diabetes mellitus
