# Supplementary material for: Immunological Characteristics in Type 2 Diabetes Mellitus Among COVID-19 Patients
Source: Front Endocrinol (Lausanne). 2021 Mar 11;12:596518. doi: 10.3389/fendo.2021.596518 (PMC7992040; doi:10.3389/fendo.2021.596518)
Supplement: Supplementary file 2 [file Table_2.docx]

**Supplementary Table 2. Comparing the counts and frequencies of immune cells on admission between survivors and non-survivors of COVID-19**

|  | Total | Non-survivors | Survivors | p value |
| --- | --- | --- | --- | --- |
|  | n=20 | n=6 | n=14 |  |
| Total T lymphocytes (%) | 70.4(56.6-76.2) | 68.5(50.6-78.0) | 70.4(58.9-74.1) | 0.934 |
| Total T lymphocytes count (10^6^/L) | 640.5(396.3-1219.3) | 379.5(69.0-638.0) | 779.5(528.0-1393.0) | 0.017 |
| Decreased, <955, n/N (%) | 14(70.0%) | 6(100.0%) | 8(57.1%) | 0.115 |
| <400, n/N (%) | 5(25.0%) | 3(50.0%) | 2(14.3%) | 0.131 |
| Total B lymphocytes (%) | 17.2(8.9-19.7) | 26.8(16.2-41.5) | 14.1(7.6-17.8) | 0.013 |
| increased, n/N (%) | 8(40.0%) | 5(83.3%) | 3(21.4%) | 0.018 |
| Total B lymphocytes count (10*6/L) | 135.5(65.5-258.3) | 128(33.7-351.2) | 135.5(68.5-236.7) | 0.934 |
| decreased, n/N (%) | 7(35.0%) | 3(50.0%) | 4(26.4%) | 0.613 |
| CD4+T cells (%) | 39.0(27.7-50.3) | 54.3(29.2-61.4) | 38.0(26.7-48.8) | 0.039 |
| CD4+T cells count (10^6^/L) | 368.5(201.0-661.8) | 271(44.0-530.5) | 410.0(203.0-776.8) | 0.161 |
| decreased, n/N (%) | 13(65.0%) | 5(83.3%) | 8(57.1%) | 0.354 |
| CD8+T cells (%) | 22.2(17.3-36.5) | 15.0(11.2-24.1) | 26.4(20.4-39.1) | 0.021 |
| CD8+T cells count (10^6^/L) | 248.0(125.8-379.0) | 57.0(24.5-125.8) | 341.0(234.7-430.5) | 0.001 |
| decreased, n/N (%) | 12(60.0%) | 6(100.0%) | 6(42.9%) | 0.042 |
| NK cells (%) | 11.4(4.6-23.67) | 3.7(2.7-12.1) | 13.6(10.3-29.2) | 0.013 |
| NK cells count (10^6^/L) | 148.0(34.3-258.8) | 22.0(3.0-54.7) | 211.5(81.5-309.5) | 0.002 |
| Decreased, <150, n/N (%) | 10(50.0%) | 6(100.0%) | 4(28.6%) | 0.011 |
| <77, n/N (%) | 8(40.0%) | 5(83.3%) | 3(21.4%) | 0.018 |
| T+B+NK(%) | 99.3(98.9-99.5) | 99.3(98.7-99.4) | 99.3(98.9-99.6) | 0.433 |
| T+B+NK(#) | 984.5(695.3-1693.5) | 615.5(96.0-974.3) | 1174.0(755.3-1765.8) | 0.032 |
| Th/Ts | 1.6(0.9-2.4) | 3.3(1.6-6.2) | 1.2(0.8-2.3) | 0.039 |
